# Influence of vegetable diets on physiological and immune responses to thermal stress in Senegalese sole (*Solea senegalensis*)

**DOI:** 10.1371/journal.pone.0194353

**Published:** 2018-03-22

**Authors:** Marta Conde-Sieira, Manuel Gesto, Sónia Batista, Fátima Linares, José L. R. Villanueva, Jesús M. Míguez, José L. Soengas, Luísa M. P. Valente

**Affiliations:** 1 CIMAR/CIIMAR, Centro Interdisciplinar de Investigação Marinha e Ambiental, Universidade do Porto, Matosinhos, Portugal; 2 Laboratorio de Fisioloxía Animal, Departamento de Bioloxía Funcional e Ciencias da Saúde, Facultade de Bioloxía and Centro Singular de Investigación Mariña-ECIMAT, Universidade de Vigo, Vigo, Spain; 3 ICBAS, Instituto de Ciências Biomédicas de Abel Salazar, Universidade do Porto, Porto, Portugal; 4 CIMA, Centro de Investigacións Mariñas, Vilanova de Arousa, Pontevedra, Spain; 5 IGAFA, Instituto Galego de formación en Acuicultura, Illa de Arousa, Pontevedra, Spain; Universitat Politècnica de València, SPAIN

## Abstract

The substitution of fish resources as ingredients for aquafeeds by those based on vegetable sources is needed to ensure aquaculture sustainability in the future. It is known that Senegalese sole (*Solea senegalensis*) accepts high dietary content of plant ingredients without altering growth or flesh quality parameters. However, scarce information is available regarding the long-term impact of vegetable diets (combining the inclusion of both vegetable protein and oils) on the stress response and immunity of this fish species. This study aims to evaluate the concomitant effect of the extended use of vegetable protein-based diets with fish oil (FO) replacement (0, 50 or 100%) by vegetable oils (VO), on the response to acute (10 min) or prolonged (4 days) stress, induced by thermal shock. Plasma levels of cortisol, glucose and lactate as well as hepatic levels of glucose, glycogen and lactate were evaluated as primary and secondary responses to stress, 6 and 18 months after feeding the experimental diets (6 and 18 MAF). The brain monoaminergic activity in telencephalon and hypothalamus, and non-specific immune parameters were also evaluated. As expected, thermal shock induced an increase in values of plasma parameters related to stress, which was more evident in acute than in prolonged stress. Stress also affected lactate levels in the liver and the values of the alternative complement pathway-ACH50 in the plasma. Dietary substitution of FO induced an effect *per se* on some parameters such as decreased hepatic glucose and glycogen levels and peroxidase activity in plasma as well enhanced serotonergic activity in brain of non-stressed fish. The results obtained in some parameters indicate that there is an interaction between the use of vegetable diets with the physiological response to thermal stress, as is the case of the hepatic lactate, serotonergic neurotransmission in brain, and the activity of ACH50 in plasma. These results suggest that the inclusion of VO in plant protein based diets point to a slightly inhibited stress response, more evident for an acute than a prolonged stress.

## Introduction

Over the last few years, a big effort has been directed to investigate the use of ingredients from vegetable sources that could substitute fishmeal (FM) and fish oil (FO) as the main constituents used in the formulation of diets for fish [1–7]. Beyond growth performance and quality flesh concerns, an appropriate diet composition is an important issue when dealing with fish health and welfare, so minimum values of essential nutrients are established for some farmed fish species [8,9].

In the presence of a stressor, the hypothalamic-pituitary-interrenal cell axis and the brain-sympathetic-chromaffin cells axis are activated inducing the release of cortisol and catecholamines, which provokes metabolic changes directed to obtain energy to help the animal overcome the threat [10,11,12]. Furthermore, the activation of brain monoaminergic systems has an important role in the recognition of the stressor and the subsequent activation of these neuroendocrine axes in fish [13,14]. The inclusion of vegetable ingredients in aquafeeds produces modifications in the fatty acid composition of the diets, inducing n-3/n-6 ratio imbalances which may affect the physiological response to stress in fish as has been reported in previous studies [15,16,17]. Particularly, cortisol release induced by stress or by ACTH stimulation is affected by the use of vegetable oils in fish which could be attributed to the different PUFA contents. Thus, for instance, α-linolenic acid (ALA) is reported to increase basal and post-stress levels of cortisol in marine fish species [15–22]. A modulation of stress-related gene expression by vegetable oils has also been reported in larvae and interrenal cells of European sea bass [17,23] and in the liver of gilthead sea bream [24].

A close interaction among neuronal, endocrine and immune systems exist during stress response in fish [25]. When the stress conditions persist for an extended time (prolonged stress), this physiological response may lose its adaptive value and be detrimental for fish health and welfare [10]. In this way, enhanced innate humoral immunity is observed under acute stress whereas a suppressive effect occurs when the stress is chronic [25]. Diets with low FM/FO contents, have low levels of n-3 long chain polyunsaturated fatty acids (LC-PUFAs), such as docosahexaenoic acid (DHA), arachidonic acid (20:4n-6) (ARA) and eicosapentaenoic acid (EPA), which are essential for marine fish. In addition, these PUFAs are precursors of the eicosanoids which have an important role in the fish health, since they can stimulate macrophages and other leucocytes against bacterial infections [15].

The effect of vegetable diets on the immune responses in fish has been assessed in previous studies with different species. The results reported so far are contradictory, as illustrated by the effects on parameters related to humoral immunity such as lysozyme activity or the alternative complement activity in serum (indicated by ACH50) in marine fish species like gilthead sea bream, Atlantic cod, European Sea bass or grouper [15,16,26–32].

Senegalese sole (*Solea senegalensis*) is a promising species in the development of South European aquaculture and its production broadly increased in the last years [1,33]. Several studies reported that the use of vegetable diets in this species has no adverse effects on growth, nutrient utilization and flesh quality as long as minimum nutrient requirements are considered [4–6,34]. Stress and immune responses of Senegalese sole has been also described in previous studies. Thus, the increased levels of cortisol, glucose and lactate in plasma have been observed in this species exposed to different stressors such as: air exposure, handling, ammonium exposure, high stocking density or thermal shock [35–40]. Enhanced serotonergic and dopaminergic activity in Senegalese sole was also observed due to the presence of stressors [37,38,41]. The effect of stress on immune parameters in Senegalese sole depends on the type and duration of the stressor agent [33]. Previous studies addressed the effect of vegetable diets (containing PP or VO) on stress and immune parameters in this species [42,43,44]. Benítez-Dorta et al. [43] reported the effect of different proportions of dietary VO inclusions on the expression of stress related genes, as the heat shock proteins (HSP) genes, obtaining a decreased expression of HSP70 in intestine and an increased HSP90AA expression in liver with the use of vegetable oils. Furthermore, an overall overexpression of immune parameters in intestine was observed with 100% of FO substitution in Senegalese sole [44]. A recent study showed that the long term feeding of Senegalese sole with practical plant protein-based diets with a partial replacement of fish oil (50%) by vegetable oils did not affect the fatty acid bioaccessibility of the fillets which could still ensure high nutritional quality for human consumption [34]. But the impact of such diets on physiological and immune responses has not been deeply assessed yet. The objective of the present study is to evaluate the effect of the long-term feeding (6 and 18 months) of Senegalese sole with practical plant protein-based diets containing increasing levels of vegetable oil on the stress and immune responses of fish, after exposure to acute or prolonged stress, induced by thermal shock. In particular, we assessed as stress response markers: cortisol, glucose and lactate levels in plasma; glucose, glycogen and lactate content in liver; serotonin (5HT), and 5-hydroxyindole-3-acetic-acid (5HIAA) contents, and 5HT/5HIAA ratio in telencephalon and hypothalamus. We also assessed lysozyme and peroxidase activities as well as the alternative complement pathway (ACH50) in plasma as markers of the humoral innate immune response.

## Materials and methods

### Ethics statement

The experimented protocols were supervised by trained scientists (following FELASA category C recommendations) and conducted according to the guidelines of protection of animals used for scientific purposes from the European directive 2010/63/UE and the Spanish Government (RD 55/2013). The institution where the experiments were carried out (IGAFA) has the approbation of Xunta de Galicia to develop the procedures (ES369010001501). The anaesthetic 2-phenoxyethanol (0.2% v/v) was used to anesthetize the animals before euthanasia by rapid decapitation.

### Experimental diets

Feed ingredients and the proximate composition of the dietary treatments are presented in Table 1. Three isoenergetic (23 KJ/g) and isolipidic (15% dry matter, DM) practical plant protein-based diets containing increasing levels of vegetable oils (0, 50 and 100% VO) were tested. All diets contained a blend of vegetal ingredients (peas, soy, wheat gluten, corn gluten, and wheat meal) as main protein source (75% plant protein sources). Diets were supplemented with selected crystalline amino acids (L-Lysine, L-Tryptophan, and DL-Methionine) to satisfy the essential amino acid requirements. The control diet (C) containing 10.6% of supplemental fish oil was compared with diets were FO was replaced by a blend of vegetal oils at a ratio of 50% (V50) or 100% (V100). The blend is a mixture of soybean (50%), rapeseed (25%) and linseed oil (25%) widely available in the market and commonly used by the feed industry. The fatty acid composition of the experimental diets is shown in Table 2. Diets were formulated and manufactured by Sparos LA (Olhão, Portugal), by means of a pilot-scale twin-screw extruder (CLEXTRAL BC45, France) with a screw diameter of 55.5 mm and temperature ranging 105–110 °C. All batches were dried in a convection oven (OP 750-UF, LTE Scientifics, United Kingdom) for 3 h at 60 °C and subsequently the oil fraction was added under vacuum conditions in a Pegasus vacuum coater (PG-10VCLAB, DINNISEN, The Netherlands). The diets were stored at 4 °C until use.

**Table 1:** Ingredients and proximate composition of the experimental diets.

	Dietary treatments		
	Control	V50	V100
*Feed ingredients (%)*			
Squid meal ^1^	15.00	15.00	15.00
Fish gelatin ^2^	2.00	2.00	2.00
Soy protein concentrate ^3^	6.00	6.00	6.00
Pea protein concentrate ^4^	15.00	15.00	15.00
Wheat gluten ^5^	16.00	16.00	16.00
Corn gluten ^6^	5.50	5.50	5.50
Wheat meal ^7^	8.00	8.00	8.00
Whole peas ^8^	5.50	5.50	5.50
Gelatinized pea meal ^9^	10.30	10.30	10.30
Fish oil ^10^	10.60	5.30	0.00
Soybean oil ^11^	0.00	2.65	5.30
Rapeseed oil ^11^	0.00	1.33	2.65
Linseed oil ^11^	0.00	1.33	2.65
Vitamin and mineral premix^12^	1.00	1.00	1.00
Binder (guar gum) ^13^	0.50	0.50	0.50
Binder (Kieselghur) ^14^	0.50	0.50	0.50
Antioxidant ^15^	0.20	0.20	0.20
Dicalcium phosphate ^16^	3.00	3.00	3.00
L-Lysine ^17^	0.20	0.20	0.20
L-Tryptophan ^18^	0.30	0.30	0.30
DL-Methionine ^19^	0.40	0.40	0.40
*Proximate composition*			
Dry matter (DM)	91.36	91.03	90.54
Crude protein (% DM)	57.83	58.43	55.15
Ash (% DM)	6.30	6.23	6.07
Crude fat (% DM)	15.29	15.24	15.06
Gross Energy (kj/g DM)	23.42	23.54	23.42

^1^ Super prime squid meal: 80% crude protein, 3.5% crude fat, Sopropêche, France.

^2^ Fish gelatin: 88% crude protein, 0.1% crude fat, LAPI Gelatine SPA, Italy.

^3^ Soycomil-P: 63% crude protein, 0.7% crude fat, ADM, The Netherlands

^4^ NUTRALYS F85F: 78% crude protein, 1% crude fat, ROQUETTE Frères, France.

^5^ VITAL: 83.7% crude protein, 1.4% crude fat, ROQUETTE Frères, France.

^6^ Corn gluten meal: 61% crude protein, 5.8% crude fat, COPAM, Portugal.

^7^ Wheat meal: 11.7% crude protein, 1.6% crude fat, Casa Lanchinha, Portugal.

^8^ Whole peas: 19.8% crude protein, 1.1% crude fat, PREMIX Lda, Portugal.

^9^ Aquatex 8071: 23.5% crude protein, 1.0% crude fat, SOTEXPRO, France.

^10^ COPPENS International, The Netherlands.

^11^ Henry Lamotte Oils GmbH, Germany.

^12^ Premix for marine fish, PREMIX Lda, Portugal. Vitamins (IU or mg kg-1 diet): DL-alpha tocopherol acetate, 100 mg; sodium menadione bisulphate, 25 mg; retinyl acetate, 20000 IU; DL-cholecalciferol, 2000 IU; thiamin, 30 mg; riboflavin, 30 mg; pyridoxine, 20 mg; cyanocobalamin, 0.1 mg; nicotinic acid, 200 mg; folic acid, 15 mg; ascorbic acid, 1000 mg; inositol, 500 mg; biotin, 3 mg; calcium panthotenate, 100 mg; choline chloride, 1000 mg, betaine, 500 mg. Minerals (g or mg kg-1 diet): cobalt carbonate, 0.65 mg; copper sulphate, 9 mg; ferric sulphate, 6 mg; potassium iodide, 0.5 mg; manganese oxide, 9.6 mg; sodium selenite, 0.01 mg; zinc sulphate,7.5 mg; sodium chloride, 400 mg; calcium carbonate, 1.86 g; excipient wheat middlings.

^13^ Guar gum HV109, SEAH International, France.

^14^ Kieselguhr (natural zeolite), LIGRANA GmbH, Germany.

^15^ Paramega PX, KEMIN EUROPE NV, Belgium.

^16^ DCP: 18% phosphorus, 23% calcium, Fosfitalia, Italy.

^17^ Lysine HCl 99%, Ajinomoto Eurolysine SAS, France.

^18^ L-Tryptophan 98%, Ajinomoto Eurolysine SAS, France.

^19^ DL-Methionine 99%, EVONIK DEGUSSA GmbH, Germany.

**Table 2:** Fatty acid composition (% of total FA) of the experimental diets.

	Treatment								
Fatty acid	CONTROL			V50			V100		
ΣSFA^a^	30.40	±	0.33	22.05	±	0.24	14.54	±	0.30
ΣMUFA^b^	27.82	±	0.41	29.05	±	0.37	29.97	±	0.12
18:2n-6 (LOA)	13.59	±	0.05	26.65	±	0.17	38.88	±	0.39
18:3n-3 (ALA)	2.21	±	0.03	8.81	±	0.12	15.11	±	0.08
20:4n-6 (ARA)	0.91	±	0.02	0.47	±	0.01	0.12	±	0.03
20:5n-3 (EPA)	11.65	±	0.25	5.87	±	0.08	0.42	±	0.07
22:5n-3 (DPA)	1.12	±	0.09	0.67	±	0.12	0.03	±	0.02
22:6n-3 (DHA)	8.19	±	0.53	4.52	±	0.19	0.86	±	0.02
ΣPUFA^c^	41.70	±	0.63	48.90	±	0.20	55.42	±	0.40
Σn-3^d^	25.87	±	0.86	21.16	±	0.05	16.43	±	0.02
Σn-6^e^	14.50	±	0.05	27.11	±	0.18	38.99	±	0.39
n-3/n-6^f^	1.78	±	0.06	0.78	±	0.01	0.42	±	0.01
DHA/EPA^g^	0.70	±	0.04	0.77	±	0.02	2.11	±	0.33
EPA/ARA^h^	12.80	±	0.54	12.54	±	0.18	3.80	±	1.22

a ΣSFA is the sum of saturated fatty acids and includes 14:0, 15:0, 16:0, 17:0, 18:0.

b ΣMUFA is the sum of mono-unsaturated fatty acids and includes 14:1, 16:1n-11, 16:1n-9, 16:1n-7, 17:1, 18:1n-9, 18:1n-7, 20:1n-9, 22:1n-11.

c ΣPUFA is the sum of polyunsaturated fatty acids and includes 16:4, 18:2n-6, 18:3n-3, 18:4n-3, 20:4n-6, 20:4n-3, 20:5n-3, 22:5n-3, 22:6n-3.

d Σn−3 is the sum of n−3 polyunsaturated fatty acids.

e Σn−6 is the sum of n−6 polyunsaturated fatty acids.

f n-3/n-6 is the ratio of Σn−3 and Σn−6.

g DHA/EPA is the ratio of docosahexaenoic fatty acid (22:6n−3) and eicosapentaenoic fatty acid (20:5n−3).

h EPA/ARA is the ratio of eicosapentaenoic fatty acid (20:5 n-3) and arachidonic acid (20:4n-6)

### Growth trial

The present experiment comprised a long-term feeding trial including a pre-fattening (0–6 months) and fattening phase (6–18 months) of Senegalese sole growing cycle. The feeding trial was conducted at the experimental facilities of IGAFA (Illa de Arousa, Spain) and juveniles of Senegalese sole were supplied by a commercial fish farm. Prior to the experiment, fish were held in quarantine for 4 weeks to adapt to the new rearing conditions. Juvenile Senegalese sole with a mean initial body weight of 13.3 ± 0.9 g were distributed among 9 fibre glass tanks (1m x 1m) (86 fish per tank, stocking density 1.14 ± 0.03 Kg.m^-2^), with a water level of 40 cm in a recirculation seawater system. Each tank was supplied with filtered, heated (20±1 °C) saltwater (30‰), at a flow rate of 2 L min^−1^. During all the experiment, dissolved O_2_, pH, and nitrogenous compounds were daily monitored and maintained at levels within limits recommended for marine fish species. Fish were exposed to an artificial photoperiod of 12 h light:12 h dark. Each experimental diet was randomly assigned to three replicate tanks. Automatic feeders delivered feeds in 4 meals/day. The daily ration was adjusted accordingly to the presence/absence of uneaten food at the bottom of each tank as previously described [45]. The size of the pellets was modified according to the fish size throughout the feeding trial. Fish were bulk-weighed monthly. After 6 and 18 months of feeding the experimental diets (6MAF and 18MAF, respectively), all fish were food deprived for 24 h, then individually weighed, and submitted to stress challenge. The average weight of the fish in those occasions was 57.09 ± 20.70 g (6MAF) and 146.24 ± 89.04 g (18MAF). Final body weight had no significant differences among the dietary experimental groups (Control = 160.63 ± 97.01 g V50 = 145.61 ± 87.01 g and V100 = 132.47 ± 83.11 g at 18MAF). The mean value of fish mortality during the growth trial was 12.54 ± 1.51%.

### Acute and prolonged stress induction

Senegalese sole fed with the different experimental diets were submitted to an acute or a prolonged stress challenge in two different occasions: after 6 and 18 months from the beginning of the feeding trial (6MAF and 18MAF, respectively). The stress challenges were carried out as follows: First of all, 12 fish of each experimental diet that were feed deprived for 24 h were removed from the 3 replicated tanks per treatment (4 fish per tank) and placed in a new tank (water at 20°C) where they were anesthetized 10 min later with 2-phenoxyethanol (0.2% v/v) in order to obtain samples of non-stressed fish to be used as control for the acute stress test. Fish were sacrificed by decapitation and samples of blood, liver, hypothalamus and telencephalon were taken and immediately frozen on dry ice. Plasma was obtained after blood centrifugation (10 min, 9000 *X* g), aliquoted, frozen in dry ice and stored at -80°C until further assays. Plasma aliquots for glucose and lactate assays were previously deproteinized with 0.6 M perchloric acid and neutralized with 1 M potassium bicarbonate.

To obtain acutely stressed fish, at each sampling point (6MAF and 18MAF), 4 fish were removed from each holding tank (a total of 12 fish per diet) where they were at 20°C, and immediately submitted to a thermal shock, by placing them in a new tank with water at 25°C. Fish were kept for 10 minutes in these stressful conditions. After stress, fish were anesthetized and sampled as detailed above. Other fish were exposed to prolonged thermal stress conditions: at each sampling point (6MAF and 18MAF), 4 fish were removed from each holding tank (a total of 12 fish per diet) where they were at 20°C and placed in new tanks with water at 25°C. Fish remained under these conditions for 4 days. Concurrently, 5 fish per treatment were placed in similar containers but with normal water temperature (20°C) during the same time (4 days) in order to have a control group comparable to the chronically stressed ones. After 4 days, 24 hour-fasted fish were anesthetized and samples were taken and stored as described above.

### Assessment of metabolite levels in plasma and liver

Plasma levels of glucose and lactate were quantified with commercial kits (Biomérieux-Ref. 61270 and Spinreact-Ref. 1001330, respectively) adapted to a microplate format. Cortisol levels were measured using a commercially available Enzyme Immunoassay Kit (Cayman, USA-Ref. 500360). In liver, a subsample of 75 mg was homogenized immediately by ultrasonic disruption with 5.5 vols of ice-cooled 0.6 M perchloric acid, and neutralized (using 1 M potassium bicarbonate). The homogenate was centrifuged (10,000 *X* g, 4 min), and the resulting supernatant was immediately frozen in dry ice and stored at -80°C until analysis. Tissue glycogen levels were assessed using the method of Keppler and Decker [46]. Glucose obtained after glycogen breakdown (after subtracting free glucose levels) was determined with a commercial kit (Biomérieux, Spain). Lactate levels in liver were also analyzed with a commercial kit (Spinreact, Spain).

### Assessment of brain monoamines and its metabolites

The contents of 5HT and 5HIAA in telencephalon and hypothalamus of Senegalese sole were analyzed by HPLC with electrochemical detection as previously described [13,37]. Tissues were weighed and homogenized by ultrasonic disruption in 0.5 ml of mobile phase used in the chromatography. Homogenates were centrifuged (16,000×*g*, 10 min) and supernatants were diluted with mobile phase prior to HPLC analysis. Data obtained were normalized by homogenate protein content. Protein was assayed in triplicate in homogenates using microplates according to the bicinchoninic acid method [47] using bovine serum albumin (Sigma) as standard.

### Assessment of humoral innate immune parameters

Plasma lysozyme activity (EU mL^−1^) was determined using a turbidimetric assay adapted to microtitre, as described by Hutchinson and Manning [48]. One lysozyme enzyme unit (EU) was defined as the amount of lysozyme that caused a decrease in absorbance per minute. Plasma peroxidase activity (EU mL^−1^) was measured following the procedure adapted to *S*. *senegalensis* by Costas et al. [49], defining that 1 unit of peroxidase produces an absorbance change of 1 OD. Alternative complement pathway (ACH50) activity was based on the lysis of rabbit red blood cells (2.8 × 108 cells mL^−1^), as reported by Sunyer and Tort [50]. ACH50 units were defined as the concentration of plasma giving 50% lysis of rabbit red blood cells. All measurements were done in triplicate on a microplate spectrophotometer ELx808 (BioTek Instrument, USA).

### Statistics

**Fig 1:**
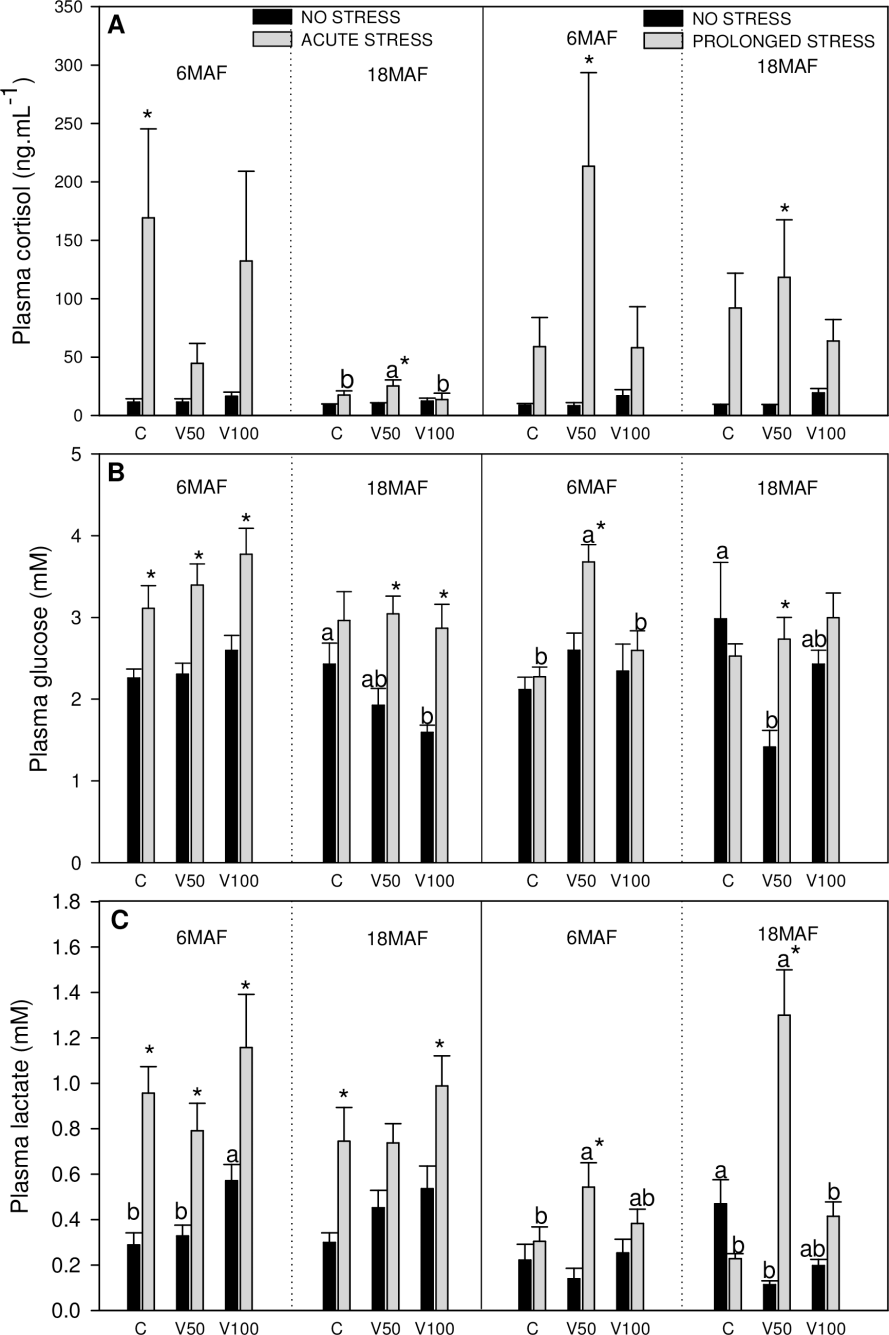
Metabolites in plasma. Cortisol (A), glucose (B) and lactate (C) levels in plasma of Senegalese sole fed the control diet (C), 50% of fish oil substitution diet (V50) or 100% of fish oil substitution diet (V100) that were non-stressed or submitted to an acute (10 min) or prolonged stress (4 days) by thermal shock (20 to 25 °C) The results of two different experiments after 6 and 18 months of feeding the experimental diets (6 and 18 MAF), are shown. Data represent mean ±SEM of 12 values. * indicates significant differences (P<0.05) with respect to the concomitant non-stressed group. Different letters indicate significant differences (P<0.05) among experimental diets under the same no stress/stress condition.

**Fig 2:**
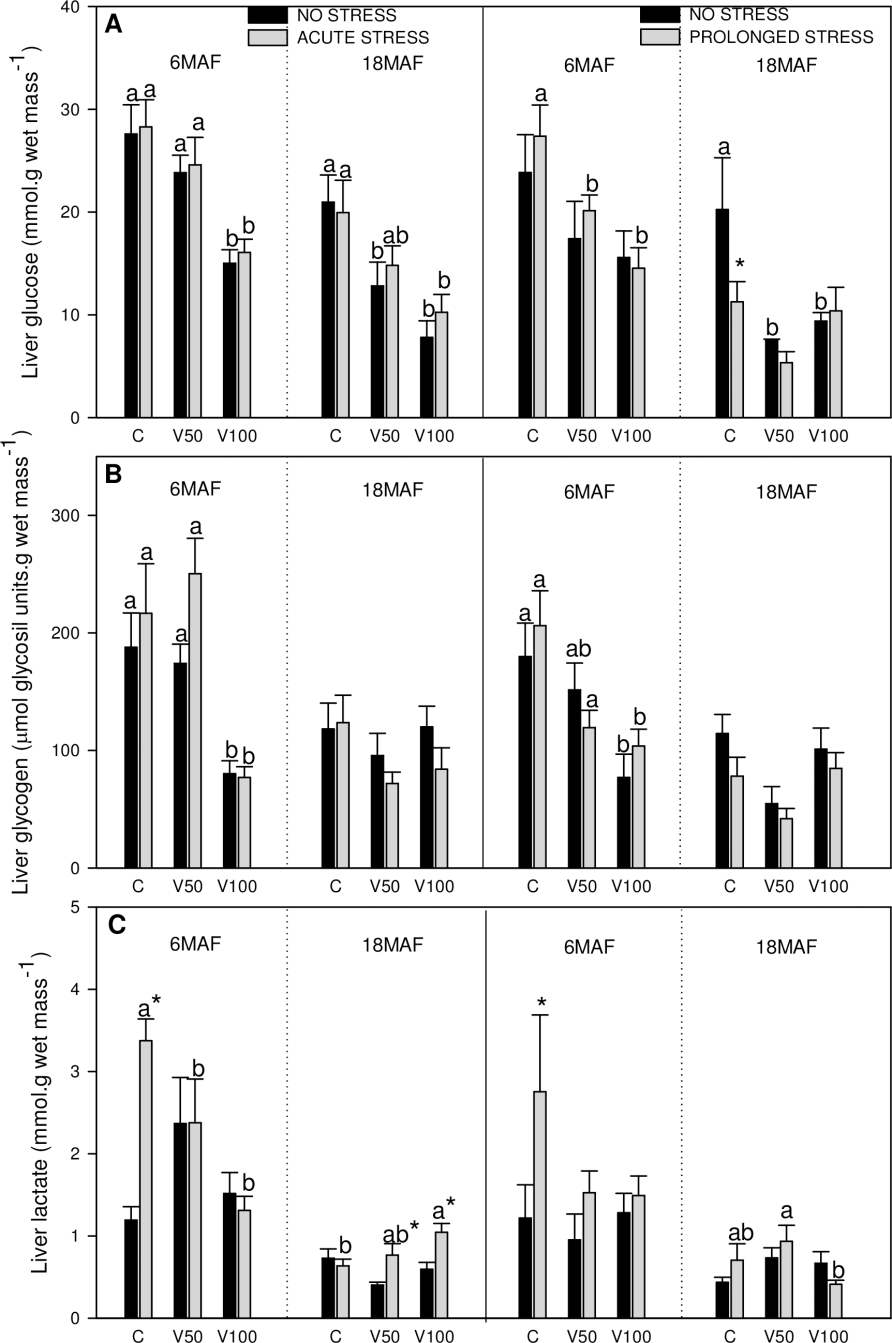
Metabolites in liver. Glucose (A), glycogen (B) and lactate (C) levels in liver of Senegalese sole fed the control diet (C), 50% of fish oil substitution diet (V50) or 100% of fish oil substitution diet (V100) that were non-stressed or submitted to an acute (10 min) or prolonged stress (4 days) by thermal shock (20 to 25 °C). For further details see Fig 1 legend.

**Fig 3:**
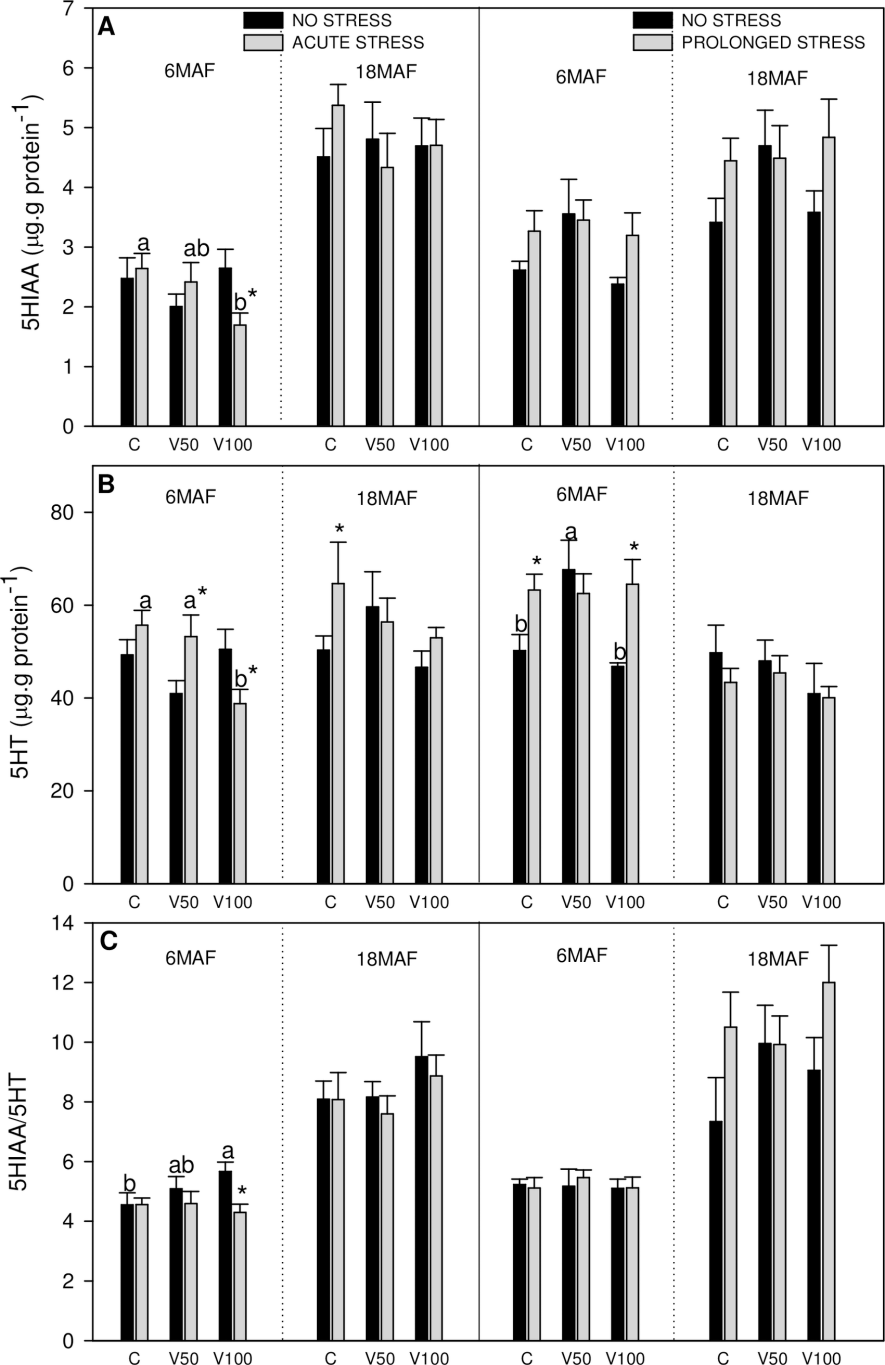
Telencephalic monoamines and its metabolites. 5HIAA (A), 5HT (B) levels or 5HIAA/5HT ratio (C) in telencephalon of Senegalese sole fed the control diet (C), 50% of fish oil substitution diet (V50) or 100% of fish oil substitution diet (V100) that were non-stressed or submitted to an acute (10 min) or prolonged stress (4 days) by thermal shock (20 to 25 °C). For further details see Fig 1 legend.

**Fig 4:**
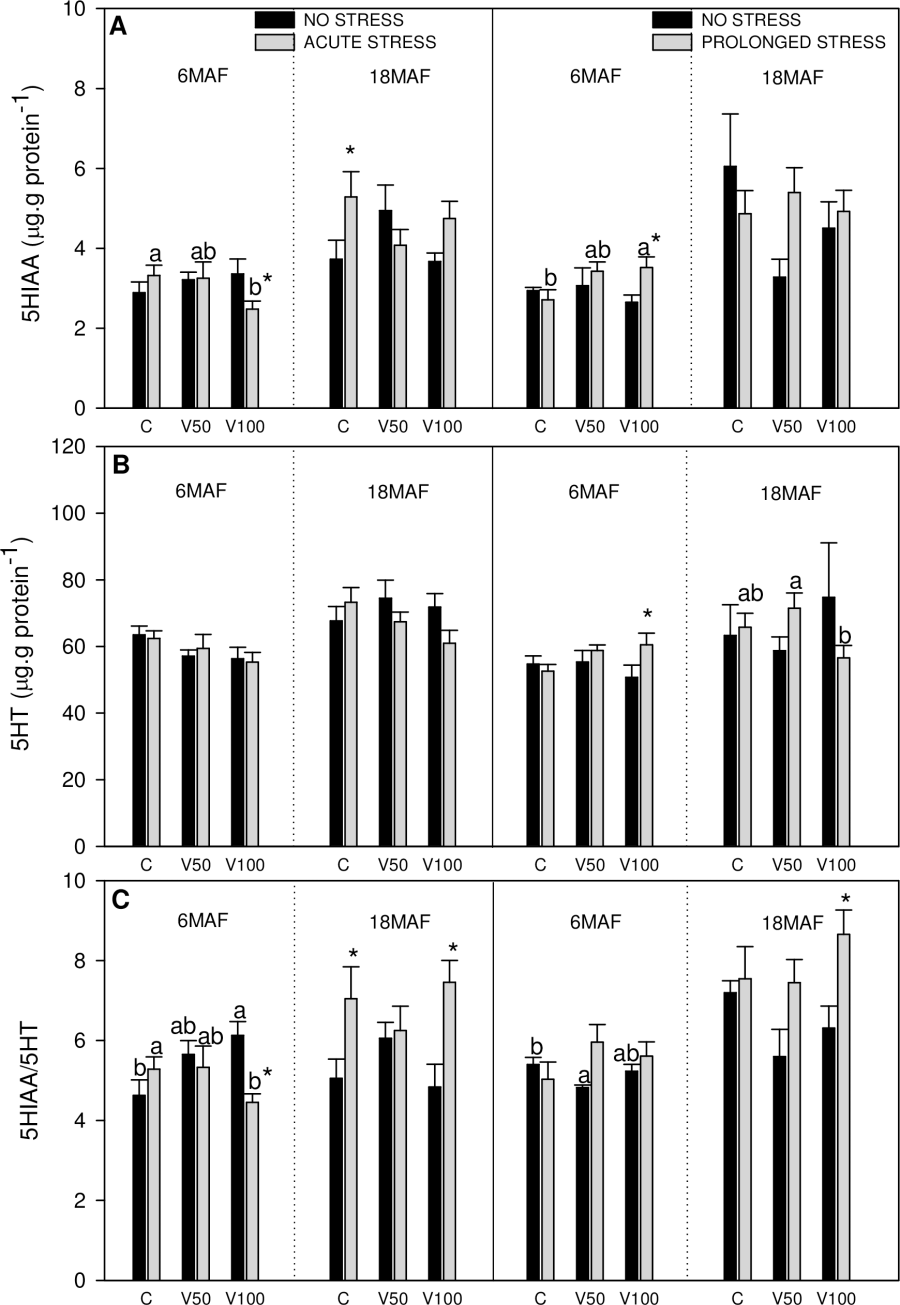
Hypothalamic monoamines and its metabolites. 5HIAA (A), 5HT (B) levels or 5HIAA/5HT ratio (C) in hypothalamus of Senegalese sole fed the control diet (C), 50% of fish oil substitution diet (V50) or 100% of fish oil substitution diet (V100) that were non-stressed or submitted to an acute (10 min) or prolonged stress (4 days) by thermal shock (20 to 25 °C). For further details see Fig 1 legend.

**Fig 5:**
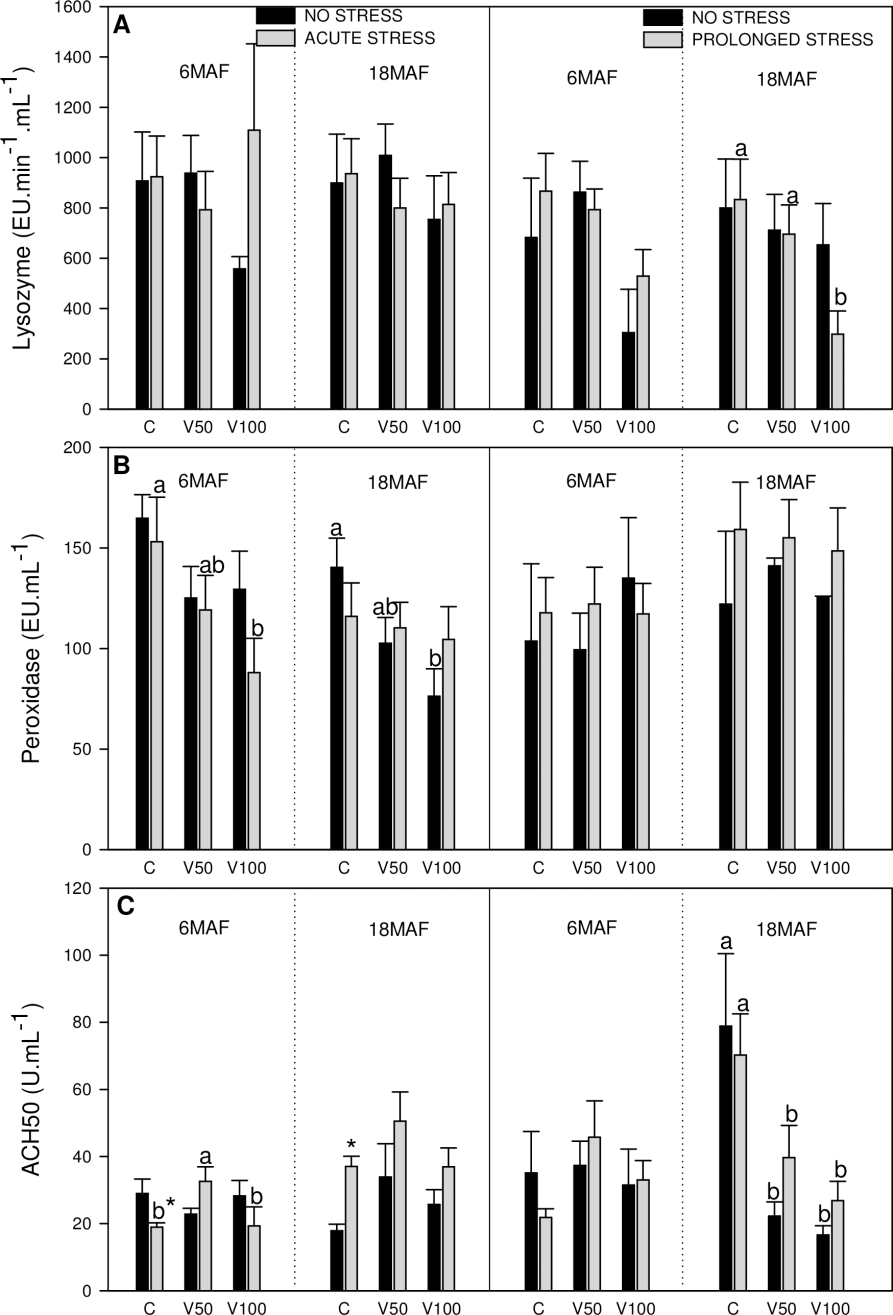
Humoral immune parameters. Levels of lysozyme (A), peroxidase (B) and ACH50 complement (C) activities in plasma of Senegalese sole fed the control diet (C), 50% of fish oil substitution diet (V50) or 100% of fish oil substitution diet (V100) that were non-stressed or submitted to an acute (10 min) or prolonged stress (4 days) by thermal shock (20 to 25 °C). For further details see Fig 1 legend.

All results are expressed as mean ± standard error of mean (SEM). Within each sampling point (6MAF and 18MAF), statistical differences were assessed with a two-way ANOVA with diet (Control, V50 and V100) and stress (no stress and stress) as main factors. Only in those cases where a significant effect was observed within a factor, post-hoc comparisons were carried out by a Student-Newman-Keuls test, and differences were considered statistically significant at *P*<0.05. When necessary, data were log transformed to fulfil the conditions of the analysis of variance. The statistical significance of the differences observed in the parameters assessed attributed to the main factors (diet and stress) and their interactions (diet x stress) in the two-way ANOVA are shown in S1 Table (acute stress) and S2 Table (prolonged stress). The significant differences resulting from post-hoc comparisons are detailed in each figure (Figs 1–5).

## Results

### Plasma and liver metabolites

The results obtained for plasmatic and hepatic metabolites related to stress response are presented in Figs 1 and 2, respectively.

Cortisol levels in plasma (Fig 1A) increased with acute stress and these increases were significant for the control at 6MAF and for V50 diet at 18MAF. Prolonged stress also induced increased cortisol levels in plasma with significant changes in V50 group at 6 and 18MAF. Plasma glucose levels (Fig 1B) decreased in non-stressed fish at 18 MAF in groups fed diets containing VO, but only V100 differed significantly from C. Glycaemia values were enhanced under acute stress conditions, although this increase was not significant in group fed C diet at 18 MAF. In control fish for prolonged stress, at 18MAF, fish fed V50 showed the lowest glucose levels. However, in the presence of prolonged stress, glucose levels in plasma increased in fish fed V50 diet. In the case of lactate values in plasma (Fig 1C) in non-stressed fish at 6MAF fed with the 100% of FO substitution (V100) showed significantly higher values than the other dietary treatments. Lactate values were enhanced in the presence of acute stress for all experimental diets. Control group of prolonged stress showed higher values than V50 group at 18MAF. When the prolonged stress was induced, V50 group showed increased values compared to other experimental diets at both sampling points.

In the liver, glucose levels (Fig 2A) decreased in groups fed V50 and V100 diets, and stress effects were only noticed at 18MAF showing lower values in fish chronically stressed and fed the C diet. In the case of glycogen levels (Fig 2B), fish fed V100 diet showed the lowest values at 6MAF for both types of stress, whereas no changes were noticed at 18MAF. Stress did not induce any changes in this parameter. Hepatic lactate levels (Fig 2C) increased significantly under stress conditions only in fish fed C diet at 6MAF. At 18MAF, acute stress induced elevated values in V50 and V100. In general, the levels of the evaluated hepatic parameters were lower at 18MAF than at 6MAF.

### Brain monoamines and its metabolites

Changes registered in cerebral monoaminergic system are presented in Fig 3 (telencephalon) and Fig 4 (hypothalamus).

Telencephalic 5HIAA values (Fig 3A) showed lower levels in acute stressed fish fed V100, at 6MAF, and this was also observed for 5HT values (Fig 3B). 5HT levels increased with acute stress in V50 at 6MAF and in C diet at 18MAF. The control fish of prolonged stress fed with V50 diet showed higher levels of 5HT than the other dietary groups. Under prolonged stress, these increased levels were observed with C and V100 diets. The ratio 5HIAA/5HT (Fig 3C) increased in non-stressed fish in parallel with the increase of FO substitution. It is worth to mention that in general the monoamine values in telencephalon were higher at 18MAF than at 6MAF, especially under acute stress conditions.

In the case of hypothalamic monoamines (Fig 4A–4C), an effect of the dietary treatment on the values of 5HIAA, 5HT and 5HIAA/5HT was also observed. At 6MAF, 5HIAA and 5HIAA/5HT were significantly reduced in fish fed V100, but only in fish under acute stress. However, under prolonged stress at 6MAF, 5HIAA values were higher in fish fed diets with higher FO substitution levels. Furthermore, acute stress induced enhanced values of 5HIAA in fish fed C diet and 5HIAA/5HT in fish fed C and V100 diets at 18MAF. Prolonged stress produced increased values of 5HIAA and 5HT at 6MAF and 5HIAA/5HT in V100 at 18MAF. In general, it was observed an effect of the dietary treatment in the parameters related to the serotonergic system in both telencephalon and hypothalamus.

### Humoral immune parameters

The results obtained for humoral immune parameters are presented in Fig 5.

Lysozyme activity in plasma (Fig 5A) was significantly lower in fish fed V100 submitted to prolonged stress at 18MAF compared to the other dietary groups. In the case of peroxidase activity in plasma (Fig 5B), under acute stress, decreasing levels were observed for the different diets with increasing FO substitution. Non-stressed fish fed V100 at 18MAF and submitted to acute stress at 6MAF, presented a significantly lower peroxidase activity than those fed the C diet. ACH50 activity (Fig 5C) was significantly reduced in acute stressed fish at 6MAF in control group, but not in V50 and V100 groups. Moreover, in acute stressed fish at 6MAF, V50 diets induced higher ACH50 activity levels than the other dietary groups. At 18MAF, ACH50 levels increased in acute stressed fish fed C diet. In control group of prolonged stress at 18 MAF, ACH50 levels were significantly lower in V50 and V100 than in C, and this effect is also observed when prolonged stress was present.

## Discussion

### Effects of thermal stress in control fish

The increase of cortisol levels in plasma is a recognized signal indicating that a response to a stressor was triggered allowing the animal to obtain enough energy to face the adverse situation [51]. Temperature is a critical factor for ectotherm animals, which need to maintain homeostasis under temperature oscillations [52]. In the present study, higher cortisol levels were observed in the control groups exposed to stress induced by higher water temperature during either 10 min (acute stress) or 4 days (prolonged stress) compared with non-stressed fish fed the control diet. The range of obtained values is similar to those reported in previous studies with Senegalese sole under stress conditions [37]. However, this increase in cortisol was only significant for acute stress at 6MAF, and not for prolonged stress, which could be due to the known negative feedback mechanisms that affect cortisol production and/or to the high interindividual variability in the plasma cortisol response after stress as a result of the presence of different copying styles in this species [10,43,53]. Previous studies have reported cortisol increments due to thermal oscillations in other fish species [54–58]. In Senegalese sole, Arjona et al. [59] did not observe cortisol changes in fish acclimated to 26°C whereas Costas et al. [60] found higher cortisol levels in this species maintained at 26 °C (with respect to controls at 18°C) but with values always within the basal range indicating that these increments could be more associated to allostatic adaptation than to a stress response [60]. In fact, these previous studies used an acclimation period (21 days) of Senegalese sole to the elevated temperatures, which could not be considered as a thermal shock. Increased cortisol levels in Senegalese sole were also found by Benítez-Dorta et al. [43] 1 hour after an acute thermal stress (increase in water temperature from 18 to 24°C within 1 hour), with values returning to basal levels 24 hours after the stress and picking again 1 week later. These results are probably responding to the mentioned negative feedback mechanisms in the cortisol production. Thus, it is important to take in account the post-stress time, as well as the quickness in the variations of temperature when interpreting the cortisol results under thermal stress. Furthermore, the present results indicate that, in general, cortisol values are lower in fish submitted to acute stress at 18MAF compared to all other groups, probably reflecting a more moderated activation of the HPI axis in bigger fish, which could reflect a diminished or delayed physiological response to stress associated with fish size. In this way, differences in stress response have also been reported in a certain number of fish species evaluated at different developmental stages [61,62].

The release of cortisol and catecholamines into the bloodstream enhances levels of plasma glucose as a result of activation of gluconeogenesis and glycogenolysis [12]. Accordingly, in the current study plasmatic glucose levels increased in control group under acute stress with respect to their concomitant non-stressed group at 6MAF but not at 18MAF. Under prolonged stress, glycaemic values did not increase probably because of the energy depletion caused by the maintenance of a prolonged stress response and the incremented metabolic rate due to the higher temperatures [10]. Accordingly, previous studies reported incremented glucose levels in plasma due to acute stress, but not under chronic stress in Senegalese sole [37,38,49]. During the response to an acute stress, there is a quick use of fuel that sometimes happens under anaerobic conditions generating lactate production [63]. Our results show significant increased levels of lactate in plasma under acute but not under prolonged stress in control groups, which is in agreement with previous studies in Senegalese sole [37,49,64].

Hepatic glucose and glycogen levels are not affected by acute stress and only hepatic glucose levels decreased in fish fed the control diet at 18MAF under prolonged stress. Previous studies indicate a decrease [37,49] or no changes [37] in liver glycogen levels in S. Sole exposed to stressors like osmotic stress, air exposure, high stocking density or poor water quality. These divergences could be attributed to the fact that hepatic parameters are highly influenced by other factors such as nutrition or temperature conditions, which could mask the effect of stress alone. In fish fed the control diet lactate values increased in liver under prolonged and acute stress as a consequence of the elevated and fast consumption of glucose under these conditions and the interchange between blood and liver.

It is known that brain monoamines play an important role in the identification of the stressing agents and the subsequent outset of the physiological stress response in fish [13,14]. Thus, it has been shown that the activity of central monoaminergic systems increases under different types of stressors, such as contaminants, handling or ammonium exposure [13,36,38] with the response of the serotonergic activity being especially consistent. The results obtained in the present study, indicate that acute stress can alter monoaminergic neurotransmission in both telencephalon and hypothalamus of Senegalese sole in agreement with previous studies [36–38,65]. Thus, increases were observed in 5HT values in telencephalon of fish fed C diets submitted to acute thermal stress. In hypothalamus, acute stress induced higher levels of 5HIAA and 5HIAA/5HT values in fish fed the C diet at 18MAF, indicating an elevated serotonergic activity under the acute stress conditions as has been reported previously in this species [38,41]. Curiously, values of 5HIAA/5HT (indicative of the activation of the serotonergic systems) are in general higher at 18MAF than at 6MAF. The reason behind those differences is not known but could relate to normal changes in brain function associated with fish development/growth. In this sense, developmental changes have been found in serotonergic brain areas of teleost fish [66].

In the present study, stress did not produce severe changes in the innate humoral immunity. Only the activity related to the alternative complement ACH50 under acute stress decreased at 6MAF and increased at 18MAF, in fish fed the control diet. The effect of stress on Senegalese sole immune parameters is controversial and depends on the origin and duration of the stressor agents [33]. Thus, stress is not always associated with immunity suppression in fish and it seems that, in the short-term, stress could have positive effects on immune systems preparing the fish for potential diseases, whereas in the long term, cortisol can exert a negative influence on immune parameters inducing a higher susceptibility of fish to pathogen attacks [25]. In the current study, neither acute nor prolonged stress induced by thermal increase exert any effect on peroxidase and lysozyme activities, although in a longer term it is known that high rearing temperatures (above 22°C) can increase susceptibility of Senegalese sole to pathogenic infections [67].

### Impact of plant origin diets on welfare parameters in absence of stress

Previous studies reported an interaction of vegetable ingredients on cortisol production in absence of stress. Thus, different contents of dietary ARA induced changes on the expression of genes related to cortisol synthesis in European sea bass larvae [23] and Senegalese sole [43]. Accordingly, basal levels of cortisol were also affected by dietary vegetable oils in Senegalese sole [16,18]. Nevertheless, no differences in cortisol levels between dietary treatments were observed in absence of stress in the present study. However, the present results indicate that at 18MAF glucose levels in plasma tended to decrease in fish fed vegetable oil diets (V50 or V100) compared to control diet in absence of acute stress. Glucose metabolism is mainly influenced by dietary composition, especially by carbohydrates, but also by lipid contents [68,69]. Borges et al. [69] demonstrated that high dietary amounts of fat can induce persistent hyperglycemia in Senegalese sole. The lower amounts of PUFA in VO based diets could also affect the metabolism of glucose in Senegalese sole. Thus, in liver, a clear effect of the substitution of FO by vegetable oil on parameters relate to glucose metabolism is evident since hepatic glucose, glycogen and lactate levels decreased significantly with increasing dietary VO levels. It seems that a prolonged use of vegetable diets leads to a hepatic energy depletion, supporting a strong interaction between lipid source and glucose metabolism in the liver of Senegalese sole. Studies addressing the effect of specific vegetable diets on glucose metabolism in fish are scarce and contradictory. In gilthead sea bream, Castro et al [70] did not find significant effects of the substitution of animal by vegetable oils based diets on carbohydrate metabolism whereas Geay et al. [71] showed elevated mRNA abundance of genes related to glucose metabolism in European sea bass fed vegetable diets. Véron et al. [72] also found differences in the expression and activity of hepatic enzymes related to glucose metabolism by the long term use of a diet based on marine resources compared with a plant based diet in rainbow trout, but such differences were more related to the differences in the starch content than in dietary sources.

Marine fish possess a low capacity to elongate LOA and ALA into long-chain FA like ARA, DHA and EPA [73] which have an important role in several biological functions related to the animal health and welfare associated with brain function. However, these deficiencies can be partially minimised providing a blend of different VO sources in fish diets [16]. The VO blend used in this experiment includes soybean oil (very rich in LOA), rapeseed oil (rich in MUFA) and LO (high content of ALA), but it still resulted in a strong reduction of omega-3 fatty acids in the VO50 and VO100 diets. To our knowledge, there are no studies evaluating the effects of plant-based diets on brain monoaminergic activity in fish. The results obtained indicate a reduced effect of the use of the dietary treatments on the parameters related to the brain serotonergic system in non-stressed fish. Thus, an incremented 5HIAA/5HT ratio was observed in telencephalon and hypothalamus of fish fed V100 after 6 months of feeding, which indicates an enhanced basal serotonergic activity in Senegalese sole caused by the total substitution of FO by VO. This incremented neurotransmission activity could be indicating a higher level of brain arousal, which could be related to a poorer welfare status of the fish. However, specific studies would be needed to evaluate the true relevance of those VO-induced changes in the serotonergic activity. There are studies in mammals that demonstrate the interaction of dietary PUFA with the monoaminergic neurotransmission, affecting the animal behaviour and health [74,75]. For instance, dietary deficiency of ALA in rats can modify FA and proteins (receptors, transporters) composition of cerebral membrane altering brain neurotransmission [75]. Furthermore, results obtained in the present study indicate that under acute stress conditions the influence of VO on the serotonergic system is higher than in the absence of stress (S1 Table).

Previous studies regarding the effect of VO on the humoral immunity in fish reported that lysozyme activities are not affected by dietary VO inclusion in agreement with the present results [15,28,29,76], whereas an incremented activity was reported by other authors [30]. Moreover, the present results indicate decreased values of ACH50 with FO replacement at 18MAF, in agreement with previous studies in other fish species [15,30,76]. This might result in a higher susceptibility of fish fed VO to pathogenic infections.

### Interaction between vegetable diets and the stress and immune responses

Some statistical interactions were found between the dietary treatments and the stress condition in the parameters evaluated in this study (S1 and S2 Tables).

The parameters related to primary and secondary stress response in plasma (cortisol, glucose, and lactate levels) present higher values in fish fed V50 diet under acute or prolonged stress. Previous studies relate dietary FA composition to the response of fish facing a stressful situation. Montero et al. [15] detected higher levels of cortisol in gilthead sea bream submitted to a stress induced by high stock density and fed a diet with 60 and 80% of FO substituted by lindseed oil. Furthermore, Alves-Martins et al. [77,78] reported increased levels of basal cortisol related to high contents of arachidonic acid (ARA) in the diet of Senegalese sole larvae. However, when dietary FO is substituted by a blend of vegetable oils and a balanced n-3 to n-6 LC-PUFA ratio is provided in the diet, no differences are found with respect to diets based on FO and even some stress effects can be prevented [16,79]. In the present study, the experimental diets present a different content of individual FA (ALA; ARA, EPA and DHA) that are known to affect cortisol production and the expression of stress related genes [17–24], and therefore could affect the degree of activation of the HPI axis in presence of stress. The mechanism through which FA exert their effect on the activation of the HPI axis could relate to an electrophysiological influence on cells of the ventromedial hypothalamus related to an inhibitory control on the activation of the HPI axis [75,80].

In liver, the interaction between the dietary treatments and the stress condition is clear in lactate levels under acute stress. Lactate values in liver increased in fish fed the control diet under prolonged and acute stress at 6MAF, but this was not observed in groups fed diets with FO replacement. However, at 18 MAF, hepatic lactate is higher in acute stressed fish fed V50 and V100. According to our results, it seems that lactate metabolism in stressed fish is influenced by the use of vegetable oils in diets, although further studies involving hepatic enzyme activities are needed to clarify the results obtained.

The interaction between the effects of stress and dietary sources is also evident in serotonergic system under acute stress in both brain areas studied. Thus, decreased values are generally observed in 5HIAA, 5HT or 5HIAA/5HT ratio in fish submitted to acute stress that were fed diets with total replacement of FO (V100). Accordingly, in mammals, alterations of the serotonergic and dopaminergic systems have been related to FA composition of the diet since contents of linolenic acid [81,82], DHA [83] or n-3 PUFAS [84,85] affect the stress response. In contrast, humoral immune parameters under thermal stress are not influenced by the content of vegetable oils in the diet.

## Conclusions

In spite of some diet-induced differences in the assessed parameters, all groups of fish were able to develop typical physiological responses (increased cortisol and increase glucose mobilization) against the stressor. The current data suggests that the interactions observed between the dietary use of vegetable diets and the physiological stress response pointed to a slightly inhibited stress response with the use of vegetable diets. For instance, the effect of thermal stress on hepatic lactate, serotonergic neurotransmission in brain, and the activity of ACH50 in plasma decrease in fish fed vegetable diets. However, V50 diet showed stress/diet interactions in plasmatic parameters indicative of stress, which could probably be attributed to the effect of the specific FA composition of this diet on HPI axis activation. Regardless of the interaction with the stress physiology, the total substitution of dietary FO by VO (V100) in Senegalese sole could affect some energetic parameters (plasmatic glucose and lactate or glucose and glycogen in liver) as well as ACH50 which could lead to higher susceptibility of fish to suffer health diseases. Further studies regarding the susceptibility of Senegalese sole to the effect of pathogenic agents related to the prolonged use of total vegetable diets would clarify this aspect.

## Supporting information

S1 Table (DOCX)

S2 Table (DOCX)
