# Supplementary material for: Influence of vegetable diets on physiological and immune responses to thermal stress in Senegalese sole (Solea senegalensis)
Source: PLoS One. 2018 Mar 22;13(3):e0194353. doi: 10.1371/journal.pone.0194353 (PMC5864020; doi:10.1371/journal.pone.0194353)
Supplement: S2 Table — (DOCX) [file pone.0194353.s002.docx]

|  |  | | 6MAF | | | 18MAF | | | |
| --- | --- | --- | --- | --- | --- | --- | --- | --- | --- |
| Parameters | | | Diet | Stress | Diet x Stress | Diet | Stress | Diet x Stress | |
| Plasma | | |  |  |  |  |  |  | |
|  | | Cortisol levels | - | <0.001 | - | - | 0.012 | - | |
|  | | Glucose levels | 0.003 | 0.025 | - | - | - | 0.042 | |
|  | | Lactate levels | - | 0.006 | - | - | - | 0.034 | |
| Liver | | |  |  |  |  |  |  | |
|  | | Glucose levels | 0.004 | - | - | 0.003 | - | - | |
|  | | Glycogen levels | <0.001 | - | - | - | - | - | |
|  | | Lactate levels | - | - | - | - | - | - | |
| Telencephalon | | |  |  |  |  |  |  | |
|  | | 5HIAA levels | - | - | - | - | - | - | |
|  | | 5HT levels | - | 0.039 | - | - | - | - | |
|  | | 5HIAA/5HT levels | - | - | - | - | - | - | |
| Hypothalamus | | |  |  |  |  |  |  | |
|  | | 5HIAA levels | - | - | - | - | - | - | |
|  | | 5HT levels | - | - | - | - | - | - | |
|  | | 5HIAA/5HT levels | - | - | - | - | 0.019 | - | |
| Humoral immune parameters | | |  |  |  |  |  |  | |
|  | | Lysozyme activity | 0.042 | - | - | - | - | - | |
|  | | Peroxidase activity | - | - | - | - | - | - | |
|  | | ACH50 activity | - | - | - | <0.001 | - | - | |
| 5HIAA, 5-hydroxyindole-3-acetic-acid  5HT, Serotonin  ACH50, Alternative complement pathway | | | | | | | | |  |

**S2 Table**. P-values obtained after two-way analysis of variance of parameters assessed in Senegalese sole after 6 and 18 months of feeding the experimental diets (6 and 18MAF). Diet (C, V50 and V100) and stress condition (PROLONGED stress or no stress) were the main factors. Diet x Stress is the first order interactions between factors. All values are significantly different unless noted by a dash
